# PD27 The Cost Effectiveness Of Non-Hospital Cardiac Rehabilitation: A Systematic Review

**DOI:** 10.1017/S0266462324002915

**Published:** 2025-01-07

**Authors:** Carmen Guirado-Fuentes, Andrea Duarte-Díaz, Estefania Herrera Ramos, Analía Abt Sacks, Lidia García-Pérez

## Abstract

**Introduction:**

Cardiac rehabilitation (CR) with physical exercise is crucial for the secondary prevention of myocardial infarction and heart failure. However, according to published studies there are differences in access to hospital-based CR depending on sex, age, ethnicity, and geographical region. An alternative is CR in non-hospital settings such as primary care, the patient’s home, or another place by means of telerehabilitation.

**Methods:**

We conducted a systematic review of full economic evaluations where non-hospital CR was compared with hospital CR in patients with ischemic heart disease or heart failure. Other eligibility criteria were model-based or clinical trial-based evaluations; studies reporting quality-adjusted life-years, years gained, or other clinical outcomes relevant to CR; and studies published in English or Spanish. Searches were conducted in June 2023 in various literature databases, including MEDLINE, Embase, CINAHL, Web of Science, INAHTA, PEDro, the Cost-Effectiveness Analysis Registry, and others. Study selection, data extraction, quality assessment, and evidence synthesis were conducted by one economist and checked by a second reviewer.

**Results:**

Nine studies were selected from the 673 references identified. Another study was identified through previous systematic reviews. Ten randomized clinical trials were included in the review. None of the studies found differences in effectiveness between hospital CR and non-hospital CR. Two studies found that non-hospital CR was less costly than hospital CR, whereas the remainder did not find any differences in costs between the two groups or were unable to demonstrate the statistical significance of any differences observed. The best conducted studies concluded that non-hospital CR was as effective as and less costly than hospital CR.

**Conclusions:**

Non-hospital CR was as cost effective as hospital CR for low- to-moderate risk patients. Based on the evidence, CR can be recommended in non-hospital settings. However, any form of CR should be evaluated after implementation because its complexity limits the generalizability of results across regions.

